# Chemometric and Physico-Chemical Characterization of Fruit and Olive Oils from Autochthonous Cultivars Grown in Aragon (Spain)

**DOI:** 10.3390/foods12040803

**Published:** 2023-02-13

**Authors:** Raquel Rey-Giménez, Sergio Vázquez Ayala, Diego Laya Reig, Ana Cristina Sánchez-Gimeno

**Affiliations:** 1 Laboratorio Agroambiental, Gobierno de Aragón, Avda, Montañana 1005, 50071 Zaragoza, Spain; 2 Instituto Agroalimentario de Aragón-IA2, Tecnología de los Alimentos, Facultad de Veterinaria, Universidad de Zaragoza, Miguel Servet 177, 50013 Zaragoza, Spain; 3 Unidad de Cultivos Leñosos, Centro de Transferencia Agroalimentaria, Gobierno de Aragón, Avda, Montañana 930, 50059 Zaragoza, Spain

**Keywords:** olive oil, chemical composition, crop year, chemometric analysis, Arbequina, fatty acids, sterols

## Abstract

Olive tree crops and the olive oil market are becoming less and less diverse due to the rise of intensive growth varieties, with the consequent loss of varietal richness provided by oils from minority and autochthonous cultivars. “Royal de Calatayud” and “Negral de Sabiñán” are two local minority cultivars in Aragon (Spain). Fruit parameters such as ripening, fresh weight, and oil yield were evaluated, as well as physico-chemical and chemical composition parameters in olive oil in comparison with “Arbequina”, a cultivar widespread in Spain and other countries. Fruits were harvested from October to December in 2017 and 2019. Chemometric analysis revealed significant varietal differences among the three cultivars. In comparison with “Arbequina”, higher oil yields were obtained in the two local cultivars. “Royal de Calatayud” has a higher oleic acid content and a greater quantity of phenolic compounds. It thus displays a better nutritional profile than “Arbequina”. This preliminary study shows that “Royal de Calatayud” could be regarded as an excellent alternative to the “Arbequina” cultivar in the parameters analyzed.

## 1. Introduction

Virgin olive oil (VOO), which comes from the fruit of the olive tree (*Olea europaea* L.), is one of the most highly appreciated vegetable oils due to its widely acknowledged nutritional and organoleptic characteristics. Its nutritional importance lies in its chemical composition. Virgin olive oil is mainly composed of triglycerides (98–99%), constituted of fatty acids. The high monounsaturated/polyunsaturated fatty acid ratio of olive oils is due to the high oleic acid content, which is the principal fatty acid (55–83%). Nutritionally relevant compounds such as biophenols, squalene, tocopherols, and sterols compose the minority fraction (1–2%). Thanks to its chemical profile, VOO is highly stable against oxidation compared to other vegetable oils, thus making it an agri-food product of great economic importance in the countries that produce it. The yield, quality, and chemical composition of virgin olive oils are strongly associated with environmental, agronomic, and genetic factors [1,2]. Knowledge of the effects of harvest date, cultivar, and crop year on the quality of virgin olive oils can provide olive farmers with powerful tools to make appropriate decisions regarding their crops.

On the other hand, the wide genetic diversity within the olive tree genus works in favor of its resistance in the face of diseases, pests, and new climatic scenarios, while also making it easier to obtain new cultivars in breeding programmes. However, the current trend in new plantations towards majority cultivars, which are not always local and therefore perhaps not adapted to the environment, detracts from the olive tree’s rich potential for the current globalized market.

In Spain, the olive sector is an industry of great social and economic importance, as this country is the world’s leading producer and exporter of olive oil [3]. Aragon, a region in the NE of Spain with 47,000 hectares dedicated to olive trees, produces 12,000 annual tonnes of olive oil [4]. It is the sixth largest olive oil-producing region in Spain. Although “Arbequina” and “Empeltre” are the cultivars with the most hectares, the Aragon region also features a wealth of local varieties [5,6,7,8]. “Royal de Calatayud” (synonyms: “Verdilla”, “Verdeña”, “Verdial”, and “Royal”) and “Negral de Sabiñán” (synonymy: “Negral”) are two of the secondary cultivars with the highest production in Aragon (7% and 1% of total hectares, respectively) [5,6,9]. As both have different names depending on the area where they are grown, it is difficult to identify them in every case [5,6,10]. No other study has previously compared “Negral de Sabiñán”, “Arbequina” and “Royal de Calatayud” cultivars with one another in the same orchard throughout the ripening period in separate crop years.

Our study’s aim was to characterize the fruit and the physico-chemical composition of the virgin olive oils of the “Royal de Calatayud” and “Negral de Sabiñán” cultivars. We also studied the influence of harvesting date, crop year, and cultivar on the parameters under analysis, using the “Arbequina” variety as a control. Chemometric analysis was applied to elucidate differences among cultivars.

## 2. Materials and Methods

### 2.1. Experimental Design

#### 2.1.1. Pedoclimatic Characteristics of the Study Area

**Figure 1:**
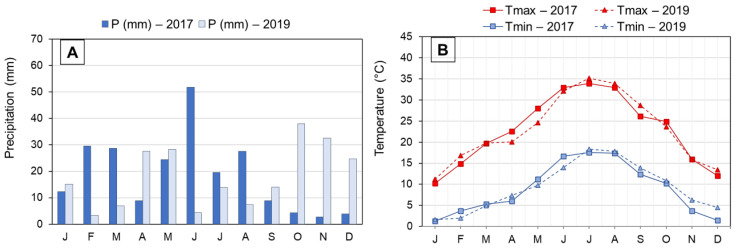
Monthly precipitation (**A**) and monthly air temperatures (maximum and minimum) (**B**) registered in Alcañiz (Teruel) during 2017 and 2019 crop seasons. Abbreviations: P, precipitations; Tmax, average maximum temperatures; Tmin, average minimum temperatures. J, January; F, February; M, March; A, April; M, May; J, June; J, July; A, August; S, September; O, October; N, November; D, December.

This study was carried out in an experimental olive orchard of the Government of Aragon located in Alcañiz, Aragon (NE Spain; 41°03′27″ N 0°08′36″ W). This region’s climate is classified as cold semi-arid (type “BSk”) according to the Köppen–Geiger climate classification [11]. It also has a marked continental, Mediterranean character. Rainfall is irregular and scarce, with May being the month with the highest precipitation and July the lowest. Average annual precipitation is 394 mm. Temperatures are extreme in winter and summer, with a wide absolute temperature range. The average temperature is 15.3 °C. Meteorological data for the two years studied are shown in Figure 1. The olive orchard was planted in 2001, in clay loam soil, with a frame of 6 × 5 m, and it was surface-irrigated.

#### 2.1.2. Olive Samples

Two local cultivars from Aragon (Spain), “Royal de Calatayud” (RdC) and “Negral de Sabiñán” (NdS), were studied in the crop years 2017 and 2019. The 2018 year was not studied due to the lack of production of “Negral de Sabiñán” and “Royal de Calatayud” cultivars because of pronounced alternate bearing behaviors. The “Arbequina” (A) cultivar was used as a control. The genotype identification of “Royal de Calatayud” was carried out by the World Olive Germplasm Bank (WOGB) (Cordoba, Spain), using the single-nucleotide polymorphism molecular marker technique (EST-SNPs).

Three olive trees per cultivar, grown under identical agronomic and pedoclimatic conditions, were harvested from October to December in 15-day intervals in each crop year. Olives (4 kg) were randomly hand-picked and immediately processed in the laboratory after harvesting.

### 2.2. Olive Fruit Analyses

Average fresh fruit weight (g) was obtained by weighing 100 fruits randomly selected from each sample. Ripeness index (RI) was determined using the methodology described by Hermoso et al. [12] based on color changes in olive skin and pulp. Oil content expressed as a percentage of dry olive paste weight was determined by first milling the olives at 3000 rpm using a hammer mill equipped with a 3 mm sieve and then analyzing the resulting olive paste with a near-infrared (NIR) analyzer (FoodScan Lab, Type 78800, Foss, UK).

### 2.3. Olive Oil Extraction

Oil extraction was performed using the Abencor^®^ system (MC2, Ingeniería y Sistemas, S.L., Seville, Spain), a laboratory mill that reproduces the industrial process of mechanical olive oil extraction. Olives were milled at 3000 rpm using a 3 mm sieve stainless hammer mill. The olive paste was subsequently malaxed at 28 °C for 30 min, and the resulting olive oil was separated by centrifugation at 3500 rpm for one minute.

The VOO thereby obtained was decanted, filtered through paper, and stored at −20 °C in amber glass bottles under a nitrogen atmosphere until analysis.

### 2.4. Olive Oil Analyses

#### 2.4.1. Physico-Chemical Quality Parameters

Free fatty acids (FFA), peroxide value (PV), and UV absorption characteristics (K_232_, K_270_, ΔK) were determined according to the analytical methods established in EU Regulation No. 2568/91 and subsequent amendments [13].

#### 2.4.2. Fatty Acid Composition

Fatty acid methyl esters (FAMEs) were prepared as described by the EU official method [13] and analyzed by gas chromatography (GC) using an Agilent chromatograph (7890N, Agilent, Santa Clara, CA, USA) equipped with a SP-2380 60 m × 0.25 mm i.d. × 0.2 µm film thickness capillary column (Supelco, Bellefonte, PA, USA). Helium was used as a carrier gas with a 1.2 mL∙min^−1^ flow. The split/spitless injector and flame ionization detector (FID) temperatures were 250 °C and 260 °C, respectively. The initial oven temperature was 170 °C, which was maintained for 30 min, followed by an increase of 5 °C∙min^−1^ up to 200 °C.

Although all regulated fatty acids were analyzed, only five of them were taken into account in this study: palmitic (C16:0), stearic (C18:0), oleic (C18:1), linoleic (C18:2), and linolenic (C18:3) acid, expressed as a percentage of FAME. Saturated (SFA), monounsaturated (MUFA), and polyunsaturated (PUFA) fatty acids, as well as the ratio of monounsaturated to polyunsaturated fatty acids (MUFA/PUFA), were also taken into consideration.

#### 2.4.3. Sterols and Triterpene Dialcohols Composition

Sterols, erythrodiol, and uvaol contents were determined following the official EU method [13]. Olive oil samples were saponified, followed by thin-layer chromatography purification on a basic silica gel plate. The recovered sterols, erythrodiol, and uvaol were silanized and subsequently analyzed using gas chromatography (6890N, Agilent, Santa Clara, CA, USA) with a CP-Sil 8CB capillary column (25 m × 0.25 mm × 0.25 µm) (Supelco, Bellefonte, PA, USA). Chromatographic working conditions were: injector and flame ionization detector (FID) temperatures, 300 °C; isothermal oven temperature, 260 °C; flow carrier gas (helium), 1 mL∙min^−1^.

#### 2.4.4. Total Phenol Content

The total phenol content in oil samples was determined by the Folin–Ciocalteau assay according to the method described by Vázquez Roncero et al. [14]. Phenolic compounds were isolated from a solution of oil (10 g) in hexane (50 mL) by triple extraction with 20 mL of a methanol/water mixture (60:40, *v*/*v*). Aqueous fractions were collected in a volumetric flask (100 mL) to obtain the total polyphenol extract. Then, 2.5 mL of Folin–Ciocalteau reagent was added to 5 mL of extract and, after 3 min, 5 mL of sodium hydroxide (6% m/m). The absorption of the solution was colorimetrically measured at 725 nm using a UV-Vis spectrophotometer (Specord 205, Analytikjena, Germany). Total phenols were expressed as mg caffeic acid∙kg^−1^ oil.

#### 2.4.5. Oxidative Stability

Oxidative stability, expressed as the oxidation induction time (h), was measured by the Rancimat method, a rapid, reliable analytical procedure [15], using a 743 Rancimat apparatus (Metrohm AG, Herisau, Switzerland). For the stability test, 3 g of oil was heated at 120 °C and an air flow of 20 L∙h^−1^ was bubbled through it.

### 2.5. Statistical Analysis

All parameters were determined in duplicate. Data were expressed as mean ± standard deviation (*n* = 2). Significant differences among samples (*p* < 0.05) were determined by univariate factorial analysis of variance (one-way ANOVA) using Tukey’s test as a multiple testing range and the t-test for independent groups. Principal component analysis (PCA) and cluster analysis were carried out using IBM SPSS Statistics 24.0 software (IBM Corp., Armonk, NY, USA). The squared Euclidean distance was used as a measure of similarity, and Ward’s method was used as a rule for clustering in the cluster analysis. Further data processing was performed with Microsoft Excel, 2016 (Microsoft Corp., Redmond, WA, USA).

## 3. Results and Discussion

### 3.1. Olive Fruit Parameters

**Figure 2:**
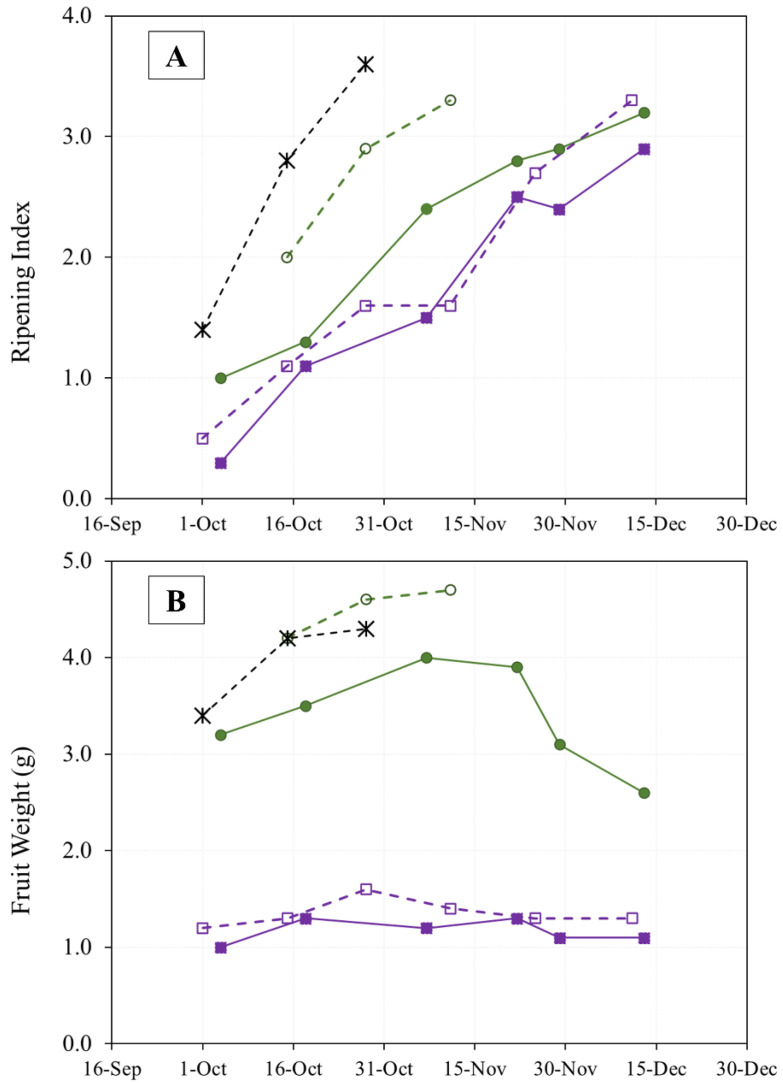
Evolution of olive fruit ripening (**A**) and fruit weight (**B**) of “Arbequina” (square; violet), “Royal de Calatayud” (circle; green), and “Negral de Sabiñán” (cross; black) harvested at different dates in 2017 (continuous line) and 2019 (dotted line).

The evolution of olive ripening in the three cultivars during two years of study is shown in Figure 2A. The thermal similarity between the sampling periods (Figure 1) in the two crop years is reflected in the speed of ripening in the “Arbequina” variety, which is also similar. The “Royal de Calatayud” cultivar ripened much more rapidly in 2019 than in 2017, perhaps due to that crop’s low yield in that year. None of the three cultivars reached a ripening index of 4 by the end of sampling.

On the other hand, the amount of rainfall recorded during the fall of 2019 (Figure 1) was higher than in 2017. This may explain the generally greater fruit weight observed in 2019, although the weight increase in the “Royal de Calatayud” cultivar was due to its low yield in that year [16]. The weights of “Royal de Calatayud” and “Negral de Sabiñán” olives in our samples were much greater than the weight of “Arbequina” (Figure 2B).

**Figure 3:**
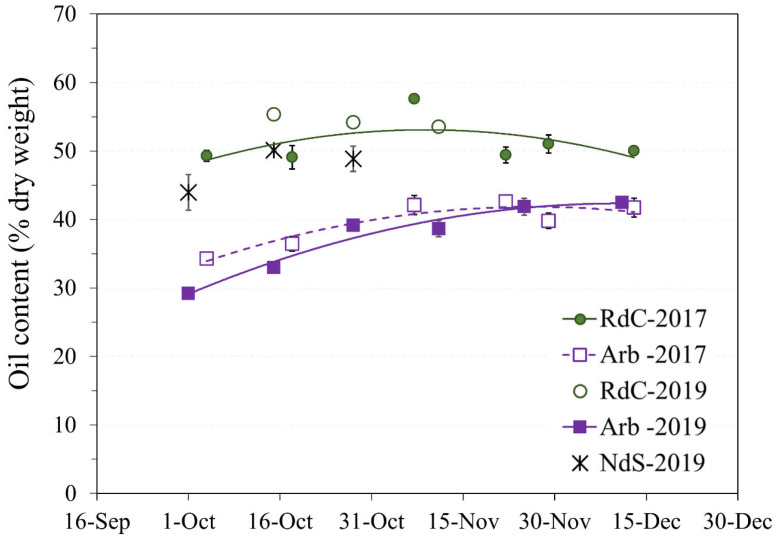
Evolution of oil content on a dry basis in the “Arbequina” (Arb; square; violet), “Royal de Calatayud” (RdC; circle; green), and “Negral de Sabiñán” (NdS; cross; black) olive oils according to harvest date in 2017 (continuous line) and 2019 (dotted line) crop years.

Olive oil content expressed as dry matter was used to avoid the influence of precipitation and frost on the study of oil accumulation or lipogenesis [17]. In general, the rate of oil accumulation progressively increased throughout the sampling period until slowing down in mid-November [16] (Figure 3).

The “Arbequina” cultivar showed a higher accumulation rate during the 2019 harvest, although the curves in the two crop years were similar. Oil content in dry matter (approximately 42%) in “Arbequina” was lower than in the two autochthonous cultivars. Other authors [18,19] have described a similar content ratio in “Arbequina”. The summer of 2019 was drier, and the autumn considerably wetter (Figure 1). This could explain the differences observed in “Arbequina” olives of similar maturity (Figure 2A) in terms of the evolution of their oil contents over the two years [20,21]. The oil content of the “Royal de Calatayud” cultivar was higher in both seasons compared to “Arbequina”. The oil content reached by the “Royal de Calatayud” cultivar by the end of the 2017 sampling was 50.5%, thus 8 percentage points higher than in “Arbequina” in the same harvest. The “Negral de Sabiñán” cultivar presented a high oil content (43.9%) at the beginning of October 2019, similar to the “Royal de Calatayud” cultivar on the exact same sampling dates. Thus, “Royal de Calatayud” and “Negral de Sabiñán” displayed high yields, which were higher than “Arbequina” in both cases.

### 3.2. Quality Parameters

All olive oils under study were classified as extra virgin olive oils (EVOO) according to the values established by EU quality criteria regulations [13]. The physico-chemical parameters we tested (acidity, peroxide value, K_232_, K_270_) showed very low values, as would be expected for oils from healthy fruits that had been processed as soon as possible after harvesting (Table 1).

### 3.3. Fatty Acids Composition

The evolutions of the fatty acid compositions of the oils derived from the two cultivars under study, compared to “Arbequina” in the course of the samplings carried out in 2017 and 2019, are detailed in Table 2. The differences in acidic composition among cultivars are significant. In previous studies, genotype had already been identified as the major source of variability in fatty acid composition [18,22,23,24,25]. Varying trends in the evolution of the main fatty acids in several cultivars have also been described according to fruit ripening and crop years [16,26,27].

In general, palmitic acid (C16:0) decreased with maturity, as other authors have likewise described [16,26,28], regardless of cultivar and year (except in “Negral de Sabiñán”, for which we had insufficient data in 2019). This fatty acid presented the highest values in “Arbequina” olive oils, with contents similar to those reported in previous studies [24,28,29]. Other authors have described C16:0 percentages as lower [30] or higher [31] for this cultivar. Regarding differences observed between the two years under study, the palmitic acid content was lower in 2017 compared to 2019 in both the “Royal de Calatayud” and “Arbequina” oils. The results from 2017 expressed as the average of all harvest samples were 11.92% for “Royal de Calatayud” and 13.78% for “Arbequina” oils. The lower temperatures along the lipogenesis period in 2017 could partially explain the lower palmitic contents observed in that year. Analogous results have been obtained for the “Picual” cultivar according to weather conditions in three crop years [16]. Stearic (C18:0) and linolenic (C18:3) fatty acids also decreased with ripening [26,28] except in oils from the “Royal de Calatayud” cultivar harvested in 2019. That crop year presented higher C18:3 contents in “Arbequina” (harvest sample average: 0.58%) and “Royal de Calatayud” (harvest sample average: 0.67%) olive oils. “Negral de Sabiñán” contained 0.60% linolenic acid in that crop year.

The trend in linoleic acid (C18:2) displayed different patterns according to cultivar and crop year. A slight increase in oils was observed throughout the “Arbequina” sampling in 2017. In the 2019 “Arbequina” oils, the rate of change was also positive, although minimal (1.62%), by the end of the sampling period. This minimal variation resulted from an initial increase in C18:2 up to the end of October, followed by a decrease until almost returning to its initial value. The variation rate of C18:2 was nil in “Royal de Calatayud” oils during 2017, but the initial points sampled in the cultivar “Royal de Calatayud”, as well as in “Negral de Sabiñán”, indicate an initial increase in linoleic acid content in 2019. However, the information obtained is insufficient to determine the trend. Hernández et al. [32] suggested that the expression of the FAD2-2 gene (OeFAD2-2) is the main responsible agent that determines linoleic acid content in the mesocarp of the olive fruit. The same study found that the water regime during the autumn season affects the expression of the FAD2-2 gene in “Arbequina”, thereby increasing linoleic acid content. Our data confirm this finding, since rainfall was more abundant in autumn 2019 than in 2017, a year with lower linoleic acid contents in “Arbequina” (sample average: 9.04%) and “Royal de Calatayud” (sample average: 6.52%). On the other hand, linoleic acid displayed high values in “Negral de Sabiñán” oils, reaching 18.63% at the end of October 2019; these data, however, could not be compared with 2017 due to a lack of samples.

Oleic acid (C18:1) in the “Arbequina” olive oils significantly increased along ripening in the two years under study. Average oleic acid content was similar in the two crop years. Nevertheless, Abenoza et al. [33] described different trends for this fatty acid in “Arbequina” oils in two harvests. The “Royal de Calatayud” oils in our study also showed a marked increase in C18:1 in 2017, although the pattern is unclear for 2019 due to insufficient data. The highest average oleic acid values (76.94%) could be observed in “Royal de Calatayud” olive oils in 2017. However, “Royal de Calatayud” oils in 2019 had a lower C18:1 content (72.06%) in contrast with the “Arbequina” oils, the contents of which were similar in the two years. The difference in C18:1 between the two crop years leads us to presume that varying climate conditions could have exerted an influence on the oleic acid content in the “Royal de Calatayud” cultivar. Certain authors [16,34,35] have affirmed that rainy summers lead to lower oleic acid content in olive oil by reducing enzymatic activity in the biosynthesis of that fatty acid. However, that observation does not coincide with our results, since we noted the highest oleic acid content in the crop year with the rainiest summer. “Negral de Sabiñán” was the cultivar with the lowest oleic acid content: an average of 64.48% in 2019. Despite limited data for “Negral de Sabiñán”, a negative trend in the evolution of oleic acid could be observed in October of that same year.

The highest MUFAs/PUFAs ratio (10.49–11.65) was observed in the “Royal de Calatayud” olive oils from 2017, and the lowest in “Negral de Sabiñán” olive oils (3.35–4.58).

### 3.4. Polyphenols and Oxidative Stability

**Figure 4:**
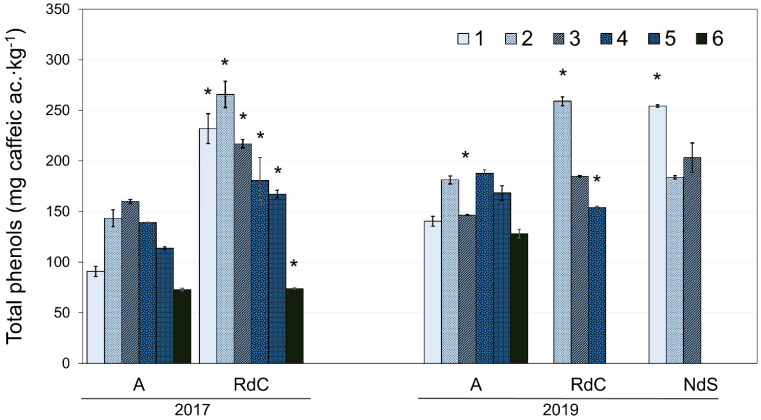
Evolution of polyphenol content for “Arbequina” (A), “Royal de Calatayud” (RdC), and “Negral de Sabiñán” (NdS) olive oils in 2017 and 2019 crop years. Harvest dates: 1 (1–4 October), 2 (16–18 October), 3 (28 October–7 November), 4 (11–22 November), 5 (25–29 November), and 6 (11–13 December). *, significant statistical differences (*p* < 0.05) among cultivars for the same crop year.

The total phenol content for each cultivar with ripening in the two crop years is presented in Figure 4. The oils generally exhibited a decrease in total polyphenol content as ripening progressed [36]. However, the Arbequina oils displayed a significant initial increase in polyphenol content until reaching a maximum towards the end of October or beginning of November, followed by a decrease, in agreement with the results described by Benito et al. [28]. Further, authors have also described this behavior in “Cornicabra”, “Chétoui”, and “Chemlali” cultivars [37,38]. Uceda et al. [22] described this variation in polyphenol content as a function of ripening with a quadratic curve whose maximum generally coincides with the maximum oil content in the fruit (see Figure 3). In 2017, oils from the “Royal de Calatayud” cultivar (74–266 mg∙kg^−1^) had a higher polyphenol content than “Arbequina” (73–160 mg∙kg^−1^). When comparing “Arbequina” oils between the two crop years, a higher phenolic content was found in 2019 (128–188 mg∙kg^−1^), a year with lower rainfall in summer but more rain in autumn. The environment, and its water availability, exerts a significant and dominant effect on phenolic concentration [23].

When comparing the phenolic contents in all three olive oils from 2019, the results show that “Negral de Sabiñán” (184–254 mg∙kg^−1^) and “Royal de Calatayud” (154–259 mg∙kg^−1^) oils had higher concentrations than the “Arbequina” cultivar.

**Figure 5:**
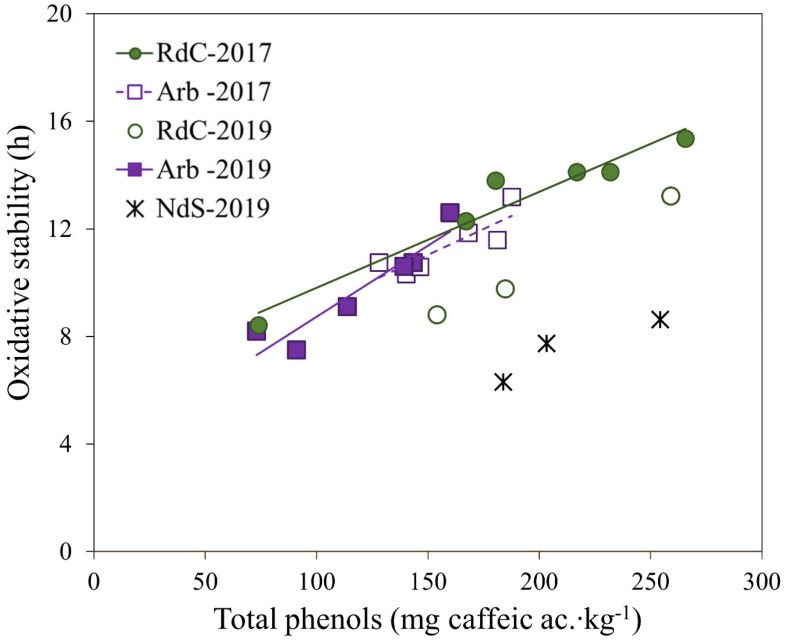
Linear regression of oxidative stability vs. total polyphenol content for “Arbequina” (Arb; square; violet), “Royal de Calatayud” (RdC; circle; green), and “Negral de Sabiñán” (NdS; cross; black) olive oil samples obtained in 2017 (solid marker) and 2019 (unfilled marker) crop years.

The three varieties displayed varying behaviors in terms of oxidative stability, which is a parameter directly related to phenolic content in olive oils [22,36,39,40]. The linear regression obtained for oxidative stability versus total polyphenol content in olive oils is shown in Figure 5. In “Arbequina” olive oils, the correlation between oxidative stability and polyphenol content was relatively high (r = 0.933, *p* < 0.01), and greater than the correlations found by other authors for the same cultivar [28,30,31,41,42]. The correlation obtained for “Royal de Calatayud” oils was lower (r = 0.818, *p* < 0.01). No relationship was found between oxidative stability and the MUFA/PUFA ratio in any of the varieties under study, in agreement with previous studies [31,43,44]. However, peroxide value did correlate negatively with stability in “Arbequina” (r = −0.772, *p* < 0.01) and “Royal de Calatayud” (r = −0.843, *p* < 0.01) (results not shown). Perhaps this correlation could be the reason for the lower stability of “Royal de Calatayud” oils compared to the 2019 “Arbequina” oils, despite their higher polyphenol content. The peroxide values of “Royal de Calatayud” oils were much higher than those of “Arbequina” in that year. The data are insufficient to yield significant correlations for oxidative stability in “Negral de Sabiñán” oils.

In 2019, the oils of the “Negral de Sabiñán” cultivar significantly presented the lowest oxidative stability (6–9 h), despite having a polyphenol content similar to the “Royal de Calatayud” oils, and even more similar to the “Arbequina” oils. The peroxide values were also low in “Negral de Sabiñán” oils (see Table 1). Perhaps the high linoleic acid content or the low MUFA/PUFA ratio were the cause of the low stability in these oils, although this could not be corroborated.

### 3.5. Minor Unsaponifiable Compounds: Sterols and Triterpenic Dialcohols

Total sterol content, individual sterol composition in relative amounts, and triterpenic diols as the sum of erythrodiol and uvaol of the analyzed olive oil samples are given in Table 3.

“Arbequina” olive oils from early ripening olives showed campesterol values above the limit (4.0%) established by European regulations [13] in both years of study (values in bold type). Other authors have also described out-of-standard campesterol contents for olive oils from the “Koroneiki” cultivar [45], for “Arbequina” and “Barnea” grown in Argentina and Australia [46,47], and for the “Cornicabra” cultivar from Spain [37,48]. The remaining parameters of the unsaponifiable fraction analyzed in all three cultivar oils comply with the limits set in the EU regulation [13].

Significant differences were found in total sterol contents among olive oils from the three cultivars. The lowest concentration of sterols was found in the oils of the “Royal de Calatayud” cultivar (1007–1360 mg∙kg^−1^), especially in the 2017 crop year, with these even approaching the acceptable limit for an oil to be considered as olive oil according to European regulation [13]. “Negral de Sabiñán” oils significantly presented the highest sterol content (1851–1947 mg∙kg^−1^). The evolution of total sterol content with ripening is not clear. In the 2017 “Arbequina” oils, after a considerable decrease at the beginning of sampling, there was an increase in sterol concentration until the initial values were reached once more. However, during the 2019 sampling, the total sterol content values remained constant. On the other hand, there was a significant increase in both crop years for the “Royal de Calatayud” olive oils and a decrease in “Negral de Sabiñán” olive oils in 2019. Certain authors have described decreases in the concentration of sterols, as these are diluted by the increase in oil that occurs during fruit ripening [36,49], although in other studies this is unclear [27,37,50]. Increases in sterol content have even been observed in direct analyses of olive pulp [51].

The main sterols in the three varieties were β-sitosterol, Δ5-avenasterol, and campesterol, making up about 95% of the total content. The significant correlations (*p* < 0.01) we found in the analyzed oils were between stigmasterol and app. β-sitosterol (r = −0.759), as well as between β-sitosterol and Δ5-avenasterol (r = −0.991); these findings coincide with those of other studies [27,50].

“Arbequina” olive oils presented the lowest β-sitosterol content (73.31–82.51%) and the highest Δ5-avenasterol content (8.11–15.76%). The values of both sterols were similar to those described in “Arbequina” by Fernández-Cuesta et al. in olive pulp [51] and Rivera del Álamo et al. in oil [48]. The Δ5-avenasterol content was lower than that reported by Gracia et al. in Aragon [29]. “Arbequina” also showed significant seasonal differences for these two sterols, with lower β-sitosterol and higher Δ5-svenasterol values in 2019 oils. The “Negral de Sabiñán” olive oils presented the highest β-sitosterol content (83.09–84.80%). The results from our complete sampling indicate that the β-sitosterol/Δ5-avenasterol ratio decreased until early November, when it stabilized until the end of sampling in all three cultivars [27]. This finding coincides with the stabilization date of olive oil content expressed as dry matter (Figure 3). Previous studies have confirmed that β-sitosterol content is minimal and Δ5-avenasterol is maximal when olives are at optimum ripeness [37,50,51,52].

Campesterol content was high in all three cultivars (3.16–4.29%), although the highest was found in “Arbequina” oils, even exceeding European regulatory limit values [13]. Campesterol decreased significantly during ripening, thereby following the same trend as β-sitosterol. Then, it reached a plateau in mid-November in “Arbequina” and “Royal de Calatayud” olive oils [45], although only in 2017 in the latter cultivar. Other authors have found no relationship between campesterol content and fruit maturity [36,37,48].

One sterol related to olive oil quality, specifically to acidity, is stigmasterol [36,53]. “Royal de Calatayud” olive oils had the highest acidity (Table 1) and stigmasterol values (1.10–1.56%). This was the only cultivar in which stigmasterol correlated significantly with acidity (r = 0.820, *p* < 0.01) [53]. The lowest stigmasterol values were in “Negral de Sabiñán” (0.57–0.79%). The results did not indicate a clear trend along ripening for this sterol.

Δ7-stigmastenol is a minor sterol in olive oil, but not in other vegetable oils such as sunflower. It is one of the markers used to verify the authenticity of olive oil and to detect adulteration with other vegetable oils [39]. The olive oils in our study had low amounts of Δ7-stigmastenol. Specifically, “Royal de Calatayud” oils showed higher percentages (0.22–0.36%), and “Negral de Sabiñán” oils the lowest (0.12–0.17%). Moreover, Δ7-stigmastenol did not display a clear trend depending on ripening, as did stigmasterol. Still, a higher amount of Δ7-stigmastenol was found at the beginning of October in the first stages of ripening in all oils under study.

Triterpenic dialcohols (erythrodiol and uvaol) are also part of the unsaponifiable fraction of olive oil, and are analyzed together with sterols to detect possible adulterations [39]. Although “Royal de Calatayud” olive oils showed high contents of those alcohols (2.79–4.35%), the sum of both lay below the maximum limit (4.5%) established by European regulations [13]. Other cultivars exhibiting high contents of these triterpenic compounds have been described [52]. At the opposite end were the “Negral de Sabiñán” oils, which had very low triterpenic alcohol contents. There was no ripening influence [49], although significant differences could be detected.

### 3.6. Chemometric Analysis

**Figure 6:**
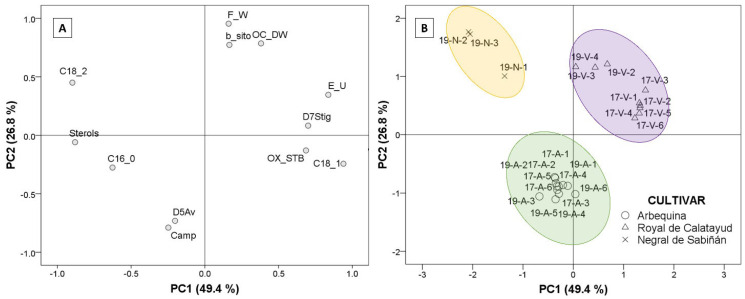
Plots of factor analysis loadings (**A**) and scores (**B**) of Principal Component Analysis (PCA) for the dataset. Cultivar: “Negral de Sabiñán” (N), “Royal de Calatayud” (V), and “Arbequina” (**A**). Sample coding: YY-C-X (YY: crop year; C: cultivar; X: harvest date). Crop years: 2017 and 2019. Harvest dates: 1 (1–4 October), 2 (16–18 October), 3 (28 October–7 November), 4 (11–22 November), 5 (25–29 November), and 6 (11–13 December).

Factor analysis extraction by Principal Component Analysis (PCA) was used to identify which of the parameters analyzed explains the differences among the oils from the three cultivars. Using Kaiser’s rule (values higher than 1), the PCA model selected three principal components that explained 88.4% of the total variance, according to the twelve studied parameters. Only the first two PCs were represented (Figure 6). The selected agronomic parameters were fresh fruit weight and oil content (% dry olive paste weight). The selected parameters in oil were principal fatty acids, such as palmitic, oleic, and linoleic acid, in addition to total sterol content, campesterol, β-sitosterol, Δ5-avenasterol, Δ7- stigmastenol, triterpenic dialcohols, and oxidative stability. Figure 6A explains the loadings corresponding to the extracted components (PC1 and PC2) according to the selected parameter. The regression method was applied to obtain the first two factor scores, which were saved as variables and subsequently represented to generate biplot (Figure 6B) via a scatter plot, labeling the cases by cultivar.

Discrimination by PCA of the three cultivars was possible, and can be studied in Figure 6. The first component (PC1; 49.4% variance) established the difference between the “Negral de Sabiñán” cultivar (negative axis) and the “Royal de Calatayud” cultivar (positive axis) according to the results. The oils from “Negral de Sabiñán” olives presented higher linoleic acid (C18_2) and sterol (Sterols) contents in comparison with “Royal de Calatayud” olive oils as a result of the negative correlations with PC1. “Royal de Calatayud” oils, on the other hand, presented higher oleic acid (C18_1), and better oxidative stability (OX_STB) via positive correlations with the first factor, in addition to higher Δ7-stigmastenol (D7Stig) and eritrodiol+uvaol (E_U) contents as unsaponifiable fraction compounds. The second principal component (PC2: 26.8% variance) clearly differentiates “Arbequina” (negative axis) from “Negral de Sabiñán” and “Royal de Calatayud” cultivars on the basis of higher campesterol (Camp) and higher Δ5-avenasterol (D5Av), but lower β-sitosterol content (b_sito). Agronomic parameters also indicated differences. Lower fruit weight (F_W) and lower oil yield (OC_DW) in “Arbequina” (negative axis) with respect to the other two cultivars were explained by the negative correlations of PC2 in fresh fruit weight and oil content (% dry olive paste weight).

**Figure 7:**
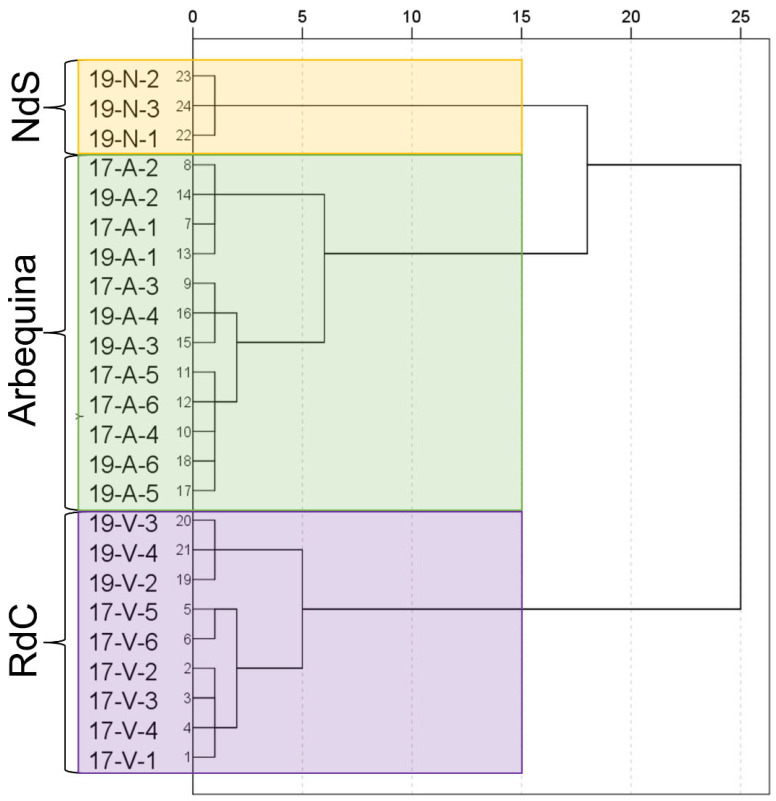
Dendrogram for the dataset. Cultivar: “Negral de Sabiñán” (N), “Royal de Calatayud” (V), and “Arbequina” (A). Sample coding: YY-C-X (YY: crop year; C: cultivar; X: harvest date). Crop years: 2017 and 2019. Harvest dates: 1 (1–4 October), 2 (16–18 October), 3 (28 October–7 November), 4 (11–22 November), 5 (25–29 November), and 6 (11–13 December).

Cluster analysis successfully allowed for a varietal classification of the cultivars on the basis of the analyzed parameters as demonstrated in the dendrogram (Figure 7). Even the influence of the crop year can be observed in “Royal de Calatayud” cultivar.

## 4. Conclusions

In this preliminary study, two minority cultivars, “Royal de Calatayud” and “Negral de Sabiñán”, were characterized with respect to “Arbequina” as regards fruit parameters and olive oil quality. The chemometric analysis results showed significant differences among cultivars. Olives from “Royal de Calatayud” and “Negral de Sabiñán” attained a greater degree of ripeness, weight, and oil yield than “Arbequina”. “Royal de Calatayud” olive oils presented the highest oxidative stability due to their better fatty acid composition, their higher oleic acid content, and their lower linoleic acid content. The polyphenol content in the oils of this cultivar was also higher than in “Arbequina”. On the other hand, “Negral de Sabiñán” olive oils, despite their high polyphenol content, were the least stable ones, perhaps due to their high linoleic acid content. The study of the unsaponifiable fraction of the oils revealed non-compliance with EU regulations only in the case of “Arbequina” oils. The campesterol content in “Arbequina” oils from unripe olives (IM ≤ 1.0) exceeded 4.0%.

In terms of nutritional value and oil yield, “Royal de Calatayud” oils are a possible attractive alternative to “Arbequina”, one of the most widespread cultivars in Spain and other countries.

Furthermore, by virtue of their specific advantages and characteristics, these monovarietal local cultivars stand out among the other virgin olive oils on the market. They could thus be reconsidered in terms of their economic potential and the variety they can introduce into the olive oil sector on a national and international level. Future experiments with more detailed studies on the responses of “Royal de Calatayud” and “Negral de Sabiñán” to varying agronomic conditions could validate our results.

## Figures and Tables

**Table 1:** Physico-chemical quality parameters of the “Arbequina” “Royal de Calatayud” and “Negral de Sabiñán” olive oils in the 2017 and 2019 crop years.

Cultivar	Harvest Date	FFA (% Oleic Acid)	PV (meq O_2_∙kg^−1^)	K_270_	K_232_
	2017				
Arbequina	4 October	0.11 ± 0.00	6.54 ± 0.00	0.091 ± 0.002	1.410 ± 0.004
	18 October	0.08 ± 0.00	4.24 ± 0.19	0.078 ± 0.000	1.339 ± 0.032
	7 November	0.13 ± 0.01	2.61 ± 0.18	0.082 ± 0.002	1.411 ± 0.001
	22 November	0.10 ± 0.00	3.96 ± 0.15	0.079 ± 0.001	1.324 ± 0.017
	29 November	0.09 ± 0.00	4.90 ± 0.14	0.082 ± 0.001	1.423 ± 0.017
	13 December	0.31 ± 0.01	5.17 ± 0.22	0.072 ± 0.001	1.345 ± 0.016
	2019				
	1 October	0.09 ± 0.00	2.36 ± 0.02	0.094 ± 0.000	1.509 ± 0.000
	15 October	0.06 ± 0.00	1.62 ± 0.00	0.083 ± 0.000	1.517 ± 0.013
	28 October	0.08 ± 0.00	1.40 ± 0.05	0.072 ± 0.001	1.505 ± 0.002
	11 November	0.08 ± 0.00	2.69 ± 0.05	0.079 ± 0.000	1.314 ± 0.004
	25 November	0.06 ± 0.00	2.15 ± 0.01	0.077 ± 0.001	1.341 ± 0.005
	11 December	0.06 ± 0.00	3.62 ± 0.04	0.074 ± 0.001	1.408 ± 0.006
	2017				
Royal de Calatayud	4 October	0.18 ± 0.01	5.47 ± 0.02	0.089 ± 0.001	1.378 ± 0.011
	18 October	0.25 ± 0.01	5.38 ± 0.09	0.090 ± 0.001	1.416 ± 0.010
	7 November	0.24 ± 0.01	4.72 ± 0.17	0.081 ± 0.000	1.343 ± 0.024
	22 November	0.23 ± 0.01	5.13 ± 0.19	0.075 ± 0.001	1.272 ± 0.036
	29 November	0.25 ± 0.01	4.50 ± 0.10	0.077 ± 0.001	1.463 ± 0.024
	13 December	0.10 ± 0.00	9.08 ± 0.14	0.073 ± 0.001	1.435 ± 0.009
	2019				
	15 October	0.41 ± 0.01	6.24 ± 0.81	0.106 ± 0.001	1.503 ± 0.020
	28 October	0.38 ± 0.00	9.64 ± 0.04	0.092 ± 0.000	1.565 ± 0.007
	11 November	-	7.67 ± 0.40	0.094 ± 0.001	1.520 ± 0.010
	2019				
Negral de Sabiñán	1 October	0.09 ± 0.00	4.11 ± 0.13	0.088 ± 0.003	1.501 ± 0.041
	15 October	0.17 ± 0.00	2.20 ± 0.02	0.083 ± 0.000	1.531 ± 0.002
	28 October	0.12 ± 0.00	1.78 ± 0.08	0.082 ± 0.001	1.460 ± 0.026

Values are mean ± standard deviation (*n* = 2). FFA = free fatty acids; PV = peroxide value. Limits established by the current European legislation for extra virgin olive oils: FFA ≤ 0.80% oleic acid; PV ≤ 20.0 meq O_2_∙kg^−1^; K_270_ ≤ 0.22; K_232_ ≤ 2.50.

**Table 2:** Evolution of fatty acid composition and indices according to harvest date for the “Arbequina”, “Royal de Calatayud” and “Negral de Sabiñán” olive oils in the 2017 and 2019 crop years.

Cultivar	Harvest Date	C16:0 (%)	C18:0 (%)	C18:1 (%)	C18:2 (%)	C18:3 (%)	SFA (%)	MUFA	PUFA	MUFA/PUFA
	2017									
Arbequina	4 October	14.32 ± 0.07 ^A;H;X^	1.91 ± 0.01 ^A;H;X^	71.84 ± 0.10 ^A;H;X^	8.76 ± 0.06 ^A;H;X^	0.62 ± 0.01 ^A;H;X^	16.98 ± 0.09 ^A;H;X^	73.65 ± 0.13 ^A;H;X^	9.38 ± 0.07 ^A;H;X^	7.82 ± 0.04 ^A;H;X^
	18 October	14.88 ± 0.08 ^B;H;X^	1.87 ± 0.01 ^B;H;X^	70.97 ± 0.10 ^B;H;X^	9.07 ± 0.06 ^B;H;X^	0.57 ± 0.01 ^B;H;X^	17.48 ± 0.09 ^B;H;X^	72.87 ± 0.13 ^B;H;X^	9.64 ± 0.07 ^B;H;X^	7.53 ± 0.04 ^B;H;X^
	7 November	14.28 ± 0.07 ^A;H;X^	1.83 ± 0.01 ^C;H;X^	71.63 ± 0.10 ^A;H;X^	9.13 ± 0.06 ^BC;H;X^	0.53 ± 0.01 ^C;H;X^	16.80 ± 0.09 ^A;H;X^	73.53 ± 0.13 ^A;H;X^	9.66 ± 0.07 ^BC;H;X^	7.58 ± 0.04 ^BC;H;X^
	22 November	13.69 ± 0.07 ^C;H;X^	1.86 ± 0.01 ^B;H;X^	72.44 ± 0.10 ^C;H;X^	9.03 ± 0.06 ^B;H;X^	0.48 ± 0.01 ^D;H;X^	16.24 ± 0.09 ^C;H;X^	74.24 ± 0.13 ^C;H;X^	9.51 ± 0.07 ^AB;H;X^	7.80 ± 0.04 ^AC;H;X^
	29 November	13.26 ± 0.07 ^D;H;X^	1.84 ± 0.0 1 ^C;H;X^	72.90 ± 0.11 ^D;H;X^	9.04 ± 0.06 ^B;H;X^	0.50 ± 0.01 ^E;H;X^	15.80 ± 0.08 ^D;H;X^	74.63 ± 0.13 ^D;H;X^	9.54 ± 0.07 ^AB;H;X^	8.30 ± 0.04 ^AC;H;X^
	13 December	12.26 ± 0.06 ^A;H;X^	1.84 ± 0.01 ^C;H;X^	73.66 ± 0.11 ^A;H;X^	9.24 ± 0.06 ^C;H;X^	0.50 ± 0.01 ^E;H;X^	14.78 ± 0.08 ^A;H;X^	75.46 ± 0.13 ^E;H;X^	9.74 ± 0.07 ^C;H;X^	7.70 ± 0.04 ^C;H;X^
	2019									
	1 October	14.76 ± 0.10 ^CA;I;X^	1.87 ± 0.01 ^A;I;X^	71.63 ± 0.06 ^A;I;X^	8.50 ± 0.04 ^A;I;X^	0.69 ± 0.01 ^A;I;X^	17.40 ± 0.12 ^A;H;X^	73.40 ± 0.06 ^A;H;X^	9.19 ± 0.05 ^A;H;X^	7.99 ± 0.04 ^A;H;X^
	15 October	15.07 ± 0.01 ^B;H;X^	1.83 ± 0.00 ^B;I;X^	70.62 ± 0.01 ^B;I;X^	9.24 ± 0.01 ^B;H;X^	0.62 ± 0.00 ^B;I;X^	17.64 ± 0.00 ^B;H;X^	72.50 ± 0.01 ^B;I;X^	9.86 ± 0.01 ^B;I;X^	7.35 ± 0.01 ^B;I;X^
	28 October	15.14 ± 0.01 ^B;I;X^	1.80 ± 0.01 ^C;I;X^	70.03 ± 0.00 ^C;I;X^	9.67 ± 0.01 ^C;I;X^	0.56 ± 0.00 ^C;I;X^	17.63 ± 0.00 ^B;I;X^	72.14 ± 0.01 ^C;I;X^	10.22 ± 0.01 ^C;I;X^	7.06 ± 0.00 ^C;I;X^
	11 November	14.20 ± 0.01 ^C;I;X^	1.81 ± 0.00 ^D;I:;X^	71.60 ± 0.01 ^A;I;X^	9.23 ± 0.01 ^B;I;X^	0.56 ± 0.00 ^C;I;X^	16.71 ± 0.00 ^C;I;X^	73.51 ± 0.00 ^D;I;X^	9.78 ± 0.01 ^D;I;X^	7.52 ± 0.01 ^D;I;X^
	25 November	13.76 ± 0.00 ^D;I^	1.79 ± 0.01 ^C;I^	71.97 ± 0.01 ^D;I^	9.37 ± 0.01 ^D;I^	0.55 ± 0.01 ^D;I^	16.22 ± 0.00 ^D;I^	73.89 ± 0.00 ^E;I^	9.91 ± 0.00 ^E;I^	7.46 ± 0.00 ^E;I^
	11 December	12.69 ± 0.01 ^E;I^	1.79 ± 0.00 ^C;I^	74.03 ± 0.03 ^E;I^	8.64 ± 0.01 ^E;I^	0.52 ± 0.00 ^E;I^	15.15 ± 0.01 ^E;I^	75.69 ± 0.02 ^F;H^	9.16 ± 0.02 ^A;I^	8.27 ± 0.02 ^F;I^
	2017									
Royal de Calatayud	4 October	13.35 ± 0.07 ^A;Y^	1.87 ± 0.01 ^A;Y^	75.17 ± 0.11 ^A;Y^	6.62 ± 0.04 ^A;Y^	0.62 ± 0.01 ^A;X^	15.97 ± 0.09 ^A;Y^	76.79 ± 0.13 ^A;Y^	7.24 ± 0.05 ^A;Y^	10.57 ± 0.06 ^A;Y^
	18 October	12.79 ± 0.07 ^B;H;Y^	1.85 ± 0.01 ^B;H;X^	75.95 ± 0.11 ^B;H;Y^	6.54 ± 0.04 ^A;H;Y^	0.59 ± 0.01 ^B;H;X^	15.36 ± 0.08 ^B;H;Y^	77.51 ± 0.13 ^B;H;Y^	7.13 ± 0.05 ^A;H;Y^	10.83 ± 0.06 ^B;H;Y^
	7 November	11.95 ± 0.06 ^C;H;Y^	1.81 ± 0.01 ^C;H;X^	76.66 ± 0.11 ^C;H;Y^	6.85 ± 0.05 ^B;H;Y^	0.57 ± 0.01 ^C;H;Y^	14.45 ± 0.08 ^C;H;Y^	78.14 ± 0.13 ^C;H;Y^	7.42 ± 0.05 ^B;H;Y^	10.49 ± 0.06 ^A;H;Y^
	22 November	11.46 ± 0.06 ^D;H;Y^	1.87 ± 0.01 ^A;H;X^	77.76 ± 0.11 ^D;H;Y^	6.22 ± 0.04 ^C;H;Y^	0.55 ± 0.01 ^D;H;Y^	14.04 ± 0.08 ^D;H;Y^	79.19 ± 0.13 ^D;H;Y^	6.77 ± 0.05 ^C;H;Y^	11.65 ± 0.06 ^C;H;Y^
	29 November	11.08 ± 0.06 ^E;Y^	1.83 ± 0.01 ^D;X^	77.80 ± 0.11 ^D;Y^	6.61 ± 0.04 ^A;Y^	0.56 ± 0.01 ^D;Y^	13.60 ± 0.07 ^E;Y^	79.22 ± 0.13 ^D;Y^	7.17 ± 0.05 ^A;Y^	11.01 ± 0.06 ^D;Y^
	13 December	10.87 ± 0.06 ^F;Y^	1.85 ± 0.01 ^B;X^	78.27 ± 0.11 ^E;Y^	6.30 ± 0.04 ^C;Y^	0.56 ± 0.01 ^CD;Y^	13.42 ± 0.07 ^E;Y^	79.69 ± 0.13 ^E;Y^	6.90 ± 0.05 ^C;Y^	11.57 ± 0.06 ^C;Y^
	2019									
	15 October	13.83 ± 0.01 ^A;I;Y^	1.92 ± 0.01 ^A;I;Y^	72.21 ± 0.01 ^A;I;Y^	8.84 ± 0.00 ^A;I;Y^	0.64 ± 0.00 ^A;I;Y^	16.40 ± 0.02 ^A;I;Y^	74.11 ± 0.01 ^A;I;Y^	9.48 ± 0.00 ^A;I;Y^	7.82 ± 0.00 ^A;I;Y^
	28 October	13.40 ± 0.02 ^B;I;Y^	1.94 ± 0.01 ^B;I;Y^	72.44 ± 0.01 ^B;I;Y^	9.09 ± 0.02 ^B;I;Y^	0.64 ± 0.01 ^A;I;Y^	15.98 ± 0.01 ^B;I;Y^	74.30 ± 0.01 ^B;I;Y^	9.72 ± 0.03 ^B;I;Y^	7.64 ± 0.02 ^B;I;Y^
	11 November	13.46 ± 0.00 ^C;I;Y^	2.06 ± 0.00 ^C;I;Y^	71.52 ± 0.02 ^C;I;Y^	9.75 ± 0.02 ^C;I;Y^	0.72 ± 0.00 ^B;I;Y^	16.23 ± 0.01 ^C;I;Y^	73.30 ± 0.02 ^C;I;Y^	10.46 ± 0.02 ^C;I;Y^	7.01 ± 0.02 ^C;I;Y^
	2019									
Negral de Sabiñán	1 October	13.63 ± 0.03 ^A;Y^	1.90 ± 0.00 ^A;Y^	67.35 ± 0.02 ^A;Y^	14.41 ± 0.01 ^A;Y^	0.61 ± 0.00 ^A;Y^	16.19 ± 0.03 ^A;Y^	68.79 ± 0.02 ^A;Y^	15.01 ± 0.01 ^A;Y^	4.58 ± 0.00 ^A;Y^
	15 October	14.07 ± 0.04 ^B;Z^	1.84 ± 0.00 ^B;Z^	63.11 ± 0.01 ^B;Z^	18.34 ± 0.02 ^B;Z^	0.59 ± 0.00 ^B;Z^	16.52 ± 0.04 ^B;Z^	64.56 ± 0.02 ^B;Z^	18.92 ± 0.02 ^B;Z^	3.41 ± 0.00 ^B;Z^
	28 October	13.92 ± 0.04 ^C;Z^	1.83 ± 0.00 ^C;Z^	62.98 ± 0.04 ^C;Z^	18.63 ± 0.00 ^C;Z^	0.60 ± 0.00 ^C;Z^	16.37 ± 0.03 ^C;Z^	64.41 ± 0.03 ^C;Z^	19.23 ± 0.00 ^C;Z^	3.35 ± 0.00 ^C;Z^

Values are mean ± standard deviation (*n* = 2). Different letters for each parameter indicate significant statistical differences (*p* < 0.05) between harvest dates for each cultivar and crop year (A–F), between crop years for each harvest date and cultivar (H–I), and between cultivars for each harvest date and crop year (X–Z). SFA = sum of saturated fatty acids; MUFA = sum of monounsatured fatty acids; PUFA = poliunsatured fatty acids.

**Table 3:** Evolution of sterol composition and triterpenic diols according to harvest date for the “Arbequina”, “Royal de Calatayud” and “Negral de Sabiñán” olive oils in the 2017 and 2019 crop years.

Cultivar	Harvest Date	Cholesterol ^†^	Campesterol ^†^	Stigmasterol ^†^	β-Sitosterol	App. β-Sitosterol ^†^	Δ5-Avenasterol	Δ7-Stigmastenol ^†^	Total sterols ^†^	E + U ^†^
	2017									
Arbequina	4 October	0.09 ± 0.01 ^AB;H;X^	4.29 ± 0.03 ^A;H;X^	0.89 ± 0.01 ^AC;H;X^	82.51 ± 0.08 ^A;H;X^	93.48 ± 0.06 ^A;H;X^	8.11 ± 0.00 ^A;H;X^	0.30 ± 0.01 ^A;H;X^	1702 ± 1 ^A;H;X^	1.65 ± 0.04 ^A;H;X^
	18 October	0.08 ± 0.01 ^AC;H;X^	4.06 ± 0.01 ^B;H;X^	0.86 ± 0.01 ^A;H;X^	80.96 ± 0.10 ^B;H;X^	93.79 ± 0.05 ^BC;H;X^	10.29 ± 0.07 ^B;H;X^	0.21 ± 0.02 ^B;H;X^	1489 ± 8 ^B;H;X^	1.61 ± 0.34 ^AC;H;X^
	7 November	0.11 ± 0.02 ^B;H;X^	3.93 ± 0.02 ^C;H;X^	0.90 ± 0.02 ^C;H;X^	77.60 ±0.08 ^C;H;X^	93.70 ± 0.09 ^B;H;X^	13.78 ± 0.01 ^C;H;X^	0.17 ± 0.00 ^CD;H;X^	1536 ± 7 ^BC;H;X^	1.62 ± 0.21 ^AC;H;X^
	22 November	0.07 ± 0.00 ^AC;H;X^	3.71 ± 0.01 ^D;H;X^	0.90 ± 0.00 ^C;H;X^	75.35 ±0.17 ^D;H;X^	93.92 ± 0.06 ^C;H;X^	15.76 ± 0.12 ^D;H;X^	0.20 ± 0.01 ^BC;H;X^	1601 ± 63 ^C;H;X^	2.06 ± 0.80 ^B;H;X^
	29 November	0.06 ± 0.00 ^C;H;X^	3.88 ± 0.01 ^E;H;X^	0.82 ± 0.01 ^D;H;X^	77.53 ±0.15 ^C;H;X^	93.94 ± 0.00 ^C;H;X^	14.35 ± 0.16 ^E;H;X^	0.16 ± 0.01 ^D;H;X^	1693 ± 9 ^A;H;X^	1.97 ± 0.02 ^AB;H;X^
	13 December	0.10 ± 0.0 ^AB;H;X^	3.83 ± 0.01 ^F;H;X^	0.88 ± 0.02 ^AC;H;X^	75.99 ± 0.50 ^E;H;X^	93.87 ± 0.04 ^C;H;X^	15.67 ± 0.14 ^D;H;X^	0.17 ± 0.03 ^CD;H;X^	1682 ± 8 ^A;H;X^	1.22 ± 0.04 ^C;H;X^
	2019									
	1 October	0.11 ± 0.02 ^A;H;X^	4.15 ± 0.02 ^A;I;X^	1.00 ± 0.02 ^A;I;X^	81.83 ± 0.19 ^A;I;X^	93.35 ± 0.10 ^A;H;X^	8.73 ± 0.00 ^A;I;X^	0.35 ± 0.02 ^A;I;X^	1407 ± 21 ^AB;I;X^	1.46 ± 0.10 ^AB;H;X^
	15 October	0.12 ± 0.04 ^A;H;X^	3.99 ± 0.02 ^B;H;X^	0.90 ± 0.02 ^B;H;X^	79.71 ± 0.42 ^B;I;X^	93.61 ± 0.16 ^BD;H;X^	10.75 ± 0.02 ^B;H;X^	0.23 ± 0.02 ^B;H;X^	1381 ± 12 ^A;I;X^	1.66 ± 0.10 ^BD;H;X^
	28 October	0.11 ± 0.05 ^A;H;X^	3.65 ± 0.01 ^C;I;X^	0.83 ± 0.02 ^CD;H;X^	73.97 ± 0.42 ^C;I;X^	93.93 ± 0.24 ^C;H;X^	17.22 ± 0.01 ^C;I;X^	0.16 ± 0.01 ^C;H;X^	1436 ± 39 ^B;H;X^	0.93 ± 0.03 ^C;I;X^
	11 November	0.12 ± 0.01 ^A;H;X^	3.88 ± 0.01 ^D;I;X^	0.85 ± 0.03 ^CD;H;X^	76.54 ± 0.28 ^D;H;X^	93.78 ± 0.29 ^D;H;X^	14.78 ± 0.02 ^D;H;X^	0.16 ± 0.01 ^C;H;X^	1415 ± 3 ^AB;H;X^	1.39 ± 0.03 ^A;H;X^
	25 November	0.12 ± 0.00 ^A;I^	3.66 ± 0.01 ^CE;H^	0.87 ± 0.01 ^BC;H^	73.31 ± 0.11 ^CE;I^	93.90 ± 0.13 ^CD;I^	18.14 ± 0.02 ^E;I^	0.14 ± 0.01 ^C;H^	1496 ± 0 ^C;I^	1.55 ± 0.03 ^AB;I^
	11 December	0.10 ± 0.01 ^A;H^	3.70 ± 0.03 ^E;I^	0.81 ± 0.00 ^D;I^	74.59 ± 0.42 ^CF;H^	93.87 ± 0.17 ^CD;H^	16.88 ± 0.03 ^C;I^	0.17 ± 0.03 ^C;H^	1400 ± 2 ^AB;I^	1.84 ± 0.12 ^D;I^
	2017									
Royal de Calatayud	4 October	0.16 ± 0.00 ^AB;Y^	3.70 ± 0.02 ^A;Y^	1.11 ± 0.01 ^A;Y^	84.90 ± 0.02 ^A;Y^	93.37 ± 0.02 ^A;X^	6.06 ± 0.01 ^A;Y^	0.36 ± 0.03 ^A;X^	1011 ± 2 ^A;Y^	3.81 ± 0.15 ^AB;Y^
	18 October	0.12 ± 0.02 ^AC;H;X^	3.43 ± 0.01 ^B;H;Y^	1.25 ± 0.02 ^B;H;Y^	84.07 ±0.07 ^B;H;Y^	93.63 ± 0.06 ^B;H;X^	7.19 ± 0.06 ^B;H;Y^	0.28 ± 0.00 ^B;H;X^	1089 ± 22 ^B;H;Y^	3.41 ± 0.46 ^AB;H;Y^
	7 November	0.18 ± 0.01 ^B;H;Y^	3.30 ± 0.02 ^C;H;Y^	1.35 ± 0.01 ^C;H;Y^	81.64 ±0.02 ^C;H;Y^	93.28 ± 0.03 ^A;H;Y^	9.50 ± 0.01 ^C;H;Y^	0.30 ± 0.02 ^B;H;Y^	1007 ± 9 ^A;H;Y^	3.25 ± 0.03 ^A;H;Y^
	22 November	0.10 ± 0.02 ^CE;H;X^	3.36 ± 0.01 ^D;H;Y^	1.15 ± 0.00 ^DA;H;Y^	81.99 ± 0.32 ^C;H;Y^	93.69 ± 0.04 ^B;H;Y^	9.72 ± 0.17 ^C;H;Y^	0.22 ± 0.02 ^C;H;X^	1263 ± 41 ^C;H;Y^	-
	29 November	0.06 ± 0.02 ^E;X^	3.42 ± 0.00 ^BE;Y^	1.17 ± 0.02 ^DE;Y^	82.63 ± 0.19 ^D;Y^	93.65 ± 0.01 ^B;Y^	9.06 ± 0.15 ^D;Y^	0.26 ± 0.02 ^BC;Y^	1360 ± 7 ^D;Y^	3.92 ± 0.23 ^B;Y^
	13 December	0.14 ± 0.02 ^ABC;X^	3.40 ± 0.03 ^DE;Y^	1.10 ± 0.02 ^A;Y^	82.57 ± 0.09 ^D;Y^	93.70 ± 0.01 ^B;X^	9.25 ± 0.01 ^D;Y^	0.27 ± 0.01 ^BC;Y^	1284 ± 15 ^C;Y^	3.91 ± 0.01 ^B;Y^
	2019									
	15 October	0.05 ± 0.02 ^A;H;X^	3.16 ± 0.02 ^A;I;Y^	1.34 ± 0.02 ^A;H;Y^	82.73 ± 0.10 ^A;I;Y^	93.76 ± 0.08 ^A;H;X^	8.38 ± 0.13 ^A;I;Y^	0.26 ± 0.01 ^A;H;X^	1185 ± 2 ^A;H;Y^	4.35 ± 0.04 ^A;H;Y^
	28 October	0.08 ± 0.02 ^A;I;X^	3.18 ± 0.03 ^A;I;Y^	1.42 ± 0.09 ^AB;H;Y^	81.50 ± 0.45 ^B;H;Y^	93.50 ± 0.22 ^AB;H;X^	9.45 ± 0.11 ^B;H;Y^	0.24 ± 0.01 ^AB;I;Y^	1240 ± 22 ^A;H;Y^	3.83 ± 0.05 ^B;I;Y^
	11 November	0.07 ± 0.03 ^A;H;X^	3.32 ± 0.02 ^B;H;Y^	1.56 ± 0.04 ^B;I;Y^	82.20 ± 0.09 ^AB;H;Y^	93.23 ± 0.00 ^B;I;Y^	8.72 ± 0.08 ^A;I;Y^	0.22 ± 0.00 ^B;H;X^	1359 ± 29 ^B;H;X^	2.79 ± 0.04 ^C;Y^
	2019									
Negral de Sabiñán	1 October	0.06 ± 0.01 ^A;X^	3.59 ± 0.01 ^A;Y^	0.57 ± 0.01 ^A;Y^	84.80 ± 0.42 ^A;Y^	94.60 ± 0.18 ^A;Y^	7.14 ± 0.19 ^A;Y^	0.17 ± 0.02 ^A;Y^	1947 ± 8 ^A;Y^	0.69 ± 0.14 ^A;Y^
	15 October	0.06 ± 0.01 ^A;X^	3.37 ± 0.02 ^B;Z^	0.58 ± 0.01 ^A;X^	84.25 ± 0.05 ^A;Z^	95.00 ± 0.10 ^A;Y^	8.24 ± 0.02 ^A;Z^	0.12 ± 0.01 ^A;Z^	1873 ± 19 ^B;Z^	0.72 ± 0.11 ^A;X^
	28 October	0.06 ± 0.01 ^A;X^	3.33 ± 0.02 ^B;Y^	0.79 ± 0.02 ^A;X^	83.09 ± 0.22 ^A;Z^	94.39 ± 0.27 ^A;Z^	8.56 ± 0.26 ^A;Z^	0.12 ± 0.05 ^A;Z^	1851 ± 13 ^B;Y^	0.80 ± 0.26 ^A;Z^

Values are mean ± standard deviation (*n* = 2). Different letters for each parameter indicate significant statistical differences (*p* < 0.05) between harvest dates for each cultivar and crop year (A–F), between crop years for each harvest date and cultivar (H–I) and between cultivars for each harvest date and crop year (X–Z). ^†^ Limits established by the current European legislation: total sterols ≥ 1000 mg/kg; cholesterol ≤ 0.5%; brassicasterol ≤ 0.1%; campesterol ≤ 4.0%; stigmasterol ≤ campesterol; Δ7-stigmastenol ≤ 0.5%; app. β-sitosterol ≥ 93%; E + U ≤ 4.5%. E + U = Sum of erythrodiol and uvaol. Values in bold type: non-compliance.

## Data Availability

Data are contained within the article.
